# Taxifolin Suppresses Inflammatory Responses of High-Glucose-Stimulated Mouse Microglia by Attenuating the TXNIP–NLRP3 Axis

**DOI:** 10.3390/nu15122738

**Published:** 2023-06-13

**Authors:** Masayo Iwasa, Hisashi Kato, Kaori Iwashita, Hajime Yamakage, Sayaka Kato, Satoshi Saito, Masafumi Ihara, Hideo Nishimura, Atsuhiko Kawamoto, Takayoshi Suganami, Masashi Tanaka, Noriko Satoh-Asahara

**Affiliations:** 1 Department of Endocrinology, Metabolism and Hypertension Research, Clinical Research Institute, National Hospital Organization Kyoto Medical Center, Kyoto 612-8555, Japan; iwasa2022@gmail.com (M.I.); hkato@mail.doshisha.ac.jp (H.K.); kaoriiwashita2012@gmail.com (K.I.); yamakage@satista.jp (H.Y.); kkatosayakaa@gmail.com (S.K.); 2 Department of Endocrinology and Metabolism, Graduate School of Medical Science, Kyoto Prefectural University of Medicine, Kyoto 602-8566, Japan; 3 Department of Neurology, National Cerebral and Cardiovascular Center, Osaka 564-8565, Japan; saitou.satoshi.43m@kyoto-u.jp (S.S.); ihara@ncvc.go.jp (M.I.); 4 Translational Research Center for Medical Innovation, Foundation for Biomedical Research and Innovation at Kobe, Kobe 650-0047, Japan; nishimura@fbri.org (H.N.); kawamoto@fbri.org (A.K.); 5 Department of Molecular Medicine and Metabolism, Research Institute of Environmental Medicine, Nagoya University, Nagoya 464-8601, Japan; suganami@riem.nagoya-u.ac.jp; 6 Department of Immunometabolism, Nagoya University Graduate School of Medicine, Nagoya 464-8601, Japan; 7 Institute of Nano-Life-Systems, Institutes of Innovation for Future Society, Nagoya University, Nagoya 464-8601, Japan; 8 Center for One Medicine Innovative Translational Research, Gifu University Institute for Advanced Study, Gifu 501-1193, Japan; 9 Department of Rehabilitation, Health Science University, Minamitsuru-gun 401-0380, Japan; 10 Department of Metabolic Syndrome and Nutritional Science, Research Institute of Environmental Medicine, Nagoya University, Nagoya 466-8550, Japan

**Keywords:** antioxidant, high glucose, inflammasome, microglia, neuroinflammation, radical scavenger, taxifolin, type 2 diabetes mellitus

## Abstract

Type 2 diabetes mellitus is associated with an increased risk of dementia, potentially through multifactorial pathologies, including neuroinflammation. Therefore, there is a need to identify novel agents that can suppress neuroinflammation and prevent cognitive impairment in diabetes. In the present study, we demonstrated that a high-glucose (HG) environment elevates the intracellular reactive oxygen species (ROS) levels and triggers inflammatory responses in the mouse microglial cell line BV-2. We further found that thioredoxin-interacting protein (TXNIP), a ROS-responsive positive regulator of the nucleotide-binding oligomerization domain (NOD)-like receptor family pyrin domain-containing 3 (NLRP3) inflammasome, was also upregulated, followed by NLRP3 inflammasome activation and subsequent interleukin-1beta (IL-1β) production in these cells. Conversely, caspase-1 was not significantly activated, suggesting the involvement of noncanonical pathways in these inflammatory responses. Moreover, our results demonstrated that taxifolin, a natural flavonoid with antioxidant and radical scavenging activities, suppressed IL-1β production by reducing the intracellular ROS levels and inhibiting the activation of the TXNIP–NLRP3 axis. These findings suggest the novel anti-inflammatory effects of taxifolin on microglia in an HG environment, which could help develop novel strategies for suppressing neuroinflammation in diabetes.

## 1. Introduction

Type 2 diabetes mellitus (T2DM) is epidemiologically associated with the risk of dementia [1,2,3]; the incidence and prevalence of dementia [4] and diabetes, respectively, is continually increasing in the elderly population [5] worldwide. Therefore, there is an urgent need to develop novel strategies for preventing and improving diabetes-related cognitive impairment.

The mechanisms underlying diabetes-related cognitive impairment have not been fully elucidated; however, growing evidence suggests the involvement of pathological processes of multifactorial pathways, such as oxidative stress, cerebrovascular damage, and central insulin resistance, in the development of neurodegeneration in the brain [6,7,8,9]. Recent preclinical studies using a mouse model of obesity/diabetes have revealed that diabetic conditions exacerbate neurotoxic mediators in the brain, including oxidative stress [10], microglial activation [10,11], and neuroinflammation [10,11], thereby resulting in cognitive impairment [10,11]. Furthermore, these disease manifestations improved along with glucose metabolism following the administration of glucagon-like peptide (GLP)-1 receptor agonists [10,11]. These results suggest that GLP-1 receptor agonists have neuroprotective effects under diabetic conditions [10,11]. Diabetes-related microglial dysfunction is reportedly implicated in the cognitive impairment of patients with T2DM [12,13,14]. Although certain anti-diabetic medications, such as GLP-1 receptor agonists, may have beneficial effects on cognitive impairment, the therapeutic efficacy of these agents has not yet been established in humans [15].

A high-glucose (HG) environment activates microglia and triggers inflammatory responses. Several molecules involved in these pathways have emerged in recent years [16]. HG induces the production of reactive oxygen species (ROS) in the microglia that subsequently activate nuclear factor-kappa B (NF-κB) to produce proinflammatory cytokines [17,18]. Mitogen-activated protein kinase (MAPK) activation is also involved in the HG-induced microglial inflammatory responses [19]. In particular, a recent study reported the role of the activation of the nucleotide-binding oligomerization domain (NOD)-like receptor family pyrin domain-containing 3 (NLRP3) inflammasome in the production of interleukin-1beta (IL-1β) in HG-stimulated microglia [20]. The activation of the NLRP3 inflammasome, which is an inflammatory multiprotein complex, promotes the formation of activated caspase-1, which releases the active form of IL-1β [21]. Furthermore, studies on renal [22] and retinal cells [23] demonstrated that elevated ROS levels attributed to diabetic conditions increased the levels of thioredoxin-interacting protein (TXNIP), a regulator of oxidative stress, which in turn activates the NLRP3 inflammasome to produce IL-1β; however, whether TXNIP is implicated in HG-induced microglial inflammation remains unclear. Thus, these components may be effective targets for suppressing neuroinflammation under diabetic conditions.

Taxifolin (dihydroquercetin) is a bioactive flavonoid with antioxidant and radical scavenging activities [24] found in various herbs and foods and exhibits various pharmacological actions [25,26,27]. We previously reported that orally administered taxifolin improves cerebral blood flow, promotes amyloid-β removal from the brain, and prevents cognitive decline [28]; it also demonstrated suppressive effects on neuroinflammation [29] in a mouse model of cerebral amyloid angiopathy. We further demonstrated the beneficial effects of taxifolin on the metabolism of obesogenic diet-fed mice; taxifolin improved glucose/lipid metabolism, obesity, and hepatic steatosis, thereby preventing nonalcoholic steatohepatitis [30]. Moreover, we recently revealed that the oral intake of taxifolin exhibits beneficial effects on the cognitive function in patients with mild cognitive impairment or mild dementia [31]. These findings suggest that taxifolin possesses therapeutic potential against diabetes-related cognitive impairment, possibly by suppressing neuroinflammation and/or improving the glucose metabolism under diabetic conditions. In this study, we focused on the in vitro action of taxifolin and investigated its effects on the inflammatory responses of microglia in an HG environment.

## 2. Materials and Methods

### 2.1. Cell Culture and Treatments

BV-2, a mouse microglial cell line, which was kindly provided by Drs. Yoneda (Kanazawa University, Ishikawa, Japan) and Hinoi (Gifu Pharmaceutical University, Gifu, Japan), was maintained in low-glucose (5.6 mmol/L) Dulbecco’s Modified Eagle’s Medium (DMEM) (Fujifilm Wako Pure Chemical Corporation, Osaka, Japan) supplemented with 10% heat-inactivated fetal bovine serum (BioWest, Bradenton, FL, USA) and 1% penicillin and streptomycin (Fujifilm Wako). The effects of taxifolin (Ametis JSC, Blagoveshchensk, Russia) on microglial activation in an HG environment were investigated as follows: Taxifolin was dissolved in DMSO at 100 mM, followed by serial dilution with the medium to obtain a final concentration of 50 μM taxifolin. The vehicle control for the taxifolin treatment was prepared in the same fashion using DMSO. Cells were pretreated for 24 h with 50 μM taxifolin or vehicle control, followed by the addition of glucose at a final concentration of 75 mM [18] or sterile distilled water as the vehicle control. After incubation for 24 h in the presence of taxifolin or a taxifolin vehicle, the cells were washed and used for subsequent analyses.

### 2.2. Cytotoxicity Assays

The cytotoxicity of taxifolin in BV-2 microglia was examined using the Cell Counting Kit-8 (Dojindo Laboratories, Kumamoto, Japan) according to the manufacturer’s instructions. Briefly, BV-2 cells were seeded in a 96-well plate at a density of 5 × 10^3^ cells/well, followed by the addition of taxifolin or a vehicle control solution. Taxifolin was dissolved in DMSO at 100 mM and serially diluted with the medium to obtain the final concentrations of interest, which had a final concentration of 0.5% DMSO. The taxifolin vehicle control was prepared in the same manner using DMSO. After the incubation of the microglia with taxifolin or the vehicle for 24 h in a CO_2_ incubator, a tetrazolium salt solution was added to each cell culture, and the plate was maintained for 2 h in the incubator. The optical density at 450 nm was subsequently measured to determine the amount of orange-colored formazan dye generated by the dehydrogenase activity in the viable cells.

### 2.3. ROS Measurement

The intracellular ROS levels were measured using the ROS Assay Kit-Highly Sensitive DCFH-DA Dye (Dojindo Laboratories) according to the manufacturer’s instructions. BV-2 cells were innoculated at a density of 3 × 10^4^ cells/well in a μ-Slide 8 Well (ibidi GmbH, Gräfelfing, Germany) and cultured as described above. After washing with Hank’s balanced salt solution (HBSS), the cells were incubated with the Highly Sensitive DCFH-DA Dye working solution for 30 min. The cells were then washed with HBSS, and fluorescence images were captured using a fluorescence microscope. Bright field images were obtained to detect cell outlines. Fluorescence intensity was measured using the ImageJ software version 1.53 k (NIH, Bethesda, MD, USA) [32].

### 2.4. Quantitative Reverse Transcription–Polymerase Chain Reaction (RT-PCR)

We extracted total RNA using an RNeasy Mini Kit (QIAGEN, Germantown, MD, USA) and synthesized first-strand cDNA using a High-Capacity RNA-to-cDNA Kit (Applied Biosystems, Waltham, MA, USA) following the manufacturer’s instructions. To examine the expression level of the gene of interest, we conducted quantitative RT-PCR using Power SYBR Green PCR Master Mix (Applied Biosystems) and a StepOnePlus Real-Time PCR System (Applied Biosystems). We determined the relative expression of each gene via the 2^−ΔΔCt^ method and used the expression levels of 18S rRNA (18S) as an internal control. The primer sequences used were as follows: mouse NADPH oxidase (Nox) 1 forward primer (5′-CGCTCCCAGCAGAAGGTCGTGATTACCAAGG-3′), reverse primer (5′-GGAGTGACCCCAATCCCTGCCCCAACCA-3′) [33]; mouse Nox2 forward primer (5′-GTGCACCATGATGAGGAGAA-3′), reverse primer (5′-TTGCAATGGTCTTGAACTCG-3′) [33]; mouse Nox4 forward primer (5′-TGTTGGGCCTAGGATTGTGTT-3′), reverse primer (5′-AGGGACCTTCTGTGATCCTCG-3′) [33] (Hokkaido System Science, Sapporo, Japan); mouse IL-1β forward primer (5′-CTGAACTCAACTGTGAAATGCCA-3′), reverse primer (5′-AAAGGTTTGGAAGCAGCCCT-3′) [34]; mouse tumor necrosis factor-alpha (TNF-α) forward primer (5′-ACCCTCACACTCAGATCATCTTC-3′), reverse primer (5′-TGGTGGTTTGCTACGACGT-3′) [35]; mouse inducible nitric oxide synthase (iNOS) forward primer (5′-CAGCTGGGCTGTACAAACCTT-3′), reverse primer (5′-CATTGGAAGTGAAGCGTTTCG-3′) [36] (Sigma-Aldrich, Tokyo, Japan); mouse TXNIP forward primer (5′-ACCACTTTCTCGGATGTTGGA-3′), reverse primer (5′-GGAAAGACAACGCCAGAAGGT-3′) (Hokkaido System Science); and mouse 18S forward primer (5′-CGATGCTCTTAGCTGAGTGT-3′), reverse primer (5′-GGTCCAAGAATTTCACCTCT-3′) [37] (Sigma-Aldrich).

### 2.5. Western Blot Analyses

We performed Western blot analyses as described previously with minor modifications [38]. Whole-cell lysates were prepared in a RIPA lysis buffer containing 20 mM HEPES (pH 7.5), 150 mM NaCl, 0.1% SDS, 1% NP-40, and 0.5% deoxycholic acid, supplemented with Halt™ Protease and Phosphatase Inhibitor Cocktail (Thermo Scientific, Waltham, MA, USA). After incubation on ice for 15 min, the homogenates were centrifuged at 14,000× g for 20 min at 4 °C, and the supernatant was stored at −80 °C for subsequent analyses. Total cellular proteins were resolved using SDS-polyacrylamide gel electrophoresis and transferred to PVDF membranes. The membranes were subsequently treated with a blocking solution (a Bullet Blocking One (Nacalai Tesque, Kyoto, Japan)) for 7 min, followed by overnight incubation at 4 °C with one of the following rabbit primary antibodies: anti-p-stress-activated protein kinase (SAPK)/Jun amino-terminal kinases (JNK) (Thr183/Tyr185) (code: #4668) (1:1000 dilution), anti-SAPK/JNK (#9252) (1:1000 dilution), anti-TXNIP (#14715) (1:1000 dilution), anti-NLRP3 (#15101) (1:1000 dilution), anti-cleaved caspase-1 (Asp296) (#89332) (1:1000 dilution), or anti-β-actin (#8457) (1:3000 dilution) (Cell Signaling Technology, Danvers, MA, USA). Immunoreactive signals were detected using an HRP-conjugated anti-rabbit IgG secondary antibody (Cell Signaling Technology) and EzWestLumi plus (ATTO, Tokyo, Japan). Gel images were captured using the ChemiDoc XRS Plus imaging system (Bio-Rad, Hercules, CA, USA), and the immunoreactive bands were quantified using ImageJ software (NIH).

### 2.6. Cytokine Measurements

BV-2 cells were seeded at a density of 1 × 10^5^ cells/well in 24-well plates, followed by incubation with the vehicle, HG, and/or taxifolin, at the final concentrations of interest. IL-1β levels in the culture supernatant were measured using a Mouse IL-1β ELISA Kit (Proteintech, Rosemont, IL, USA), according to the manufacturer’s instructions.

### 2.7. Statistical Analysis

All the data are expressed as the mean ± standard error of mean (SEM). The means between groups were compared using the one-way analysis of variance (ANOVA) with Tukey’s post hoc tests for pairwise comparisons using GraphPad Prism version 9 (GraphPad Software, San Diego, CA, USA). *p* < 0.05 was considered to be statistically significant.

## 3. Results

### 3.1. Cytotoxicity of Taxifolin on the Mouse Microglial Cell Line

**Figure 1:**
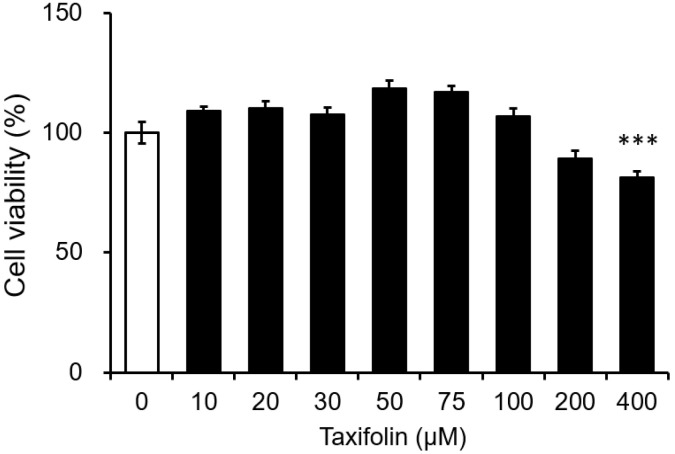
Cytotoxic effects of taxifolin on BV-2 microglia. Cells were incubated with taxifolin for 24 h at the indicated final concentrations. The cytotoxicity was determined via a colorimetric assay based on the dehydrogenase activity. Data are expressed as the mean ± SEM (*n* = 3; three independent experiments were performed). *** *p* < 0.001 vs. the vehicle control.

First, we investigated the cytotoxic effects of taxifolin on BV-2 microglia. Taxifolin exhibited no significant cytotoxicity against BV-2 cells at concentrations up to 200 μM, whereas 400 μM taxifolin significantly reduced the viability of BV-2 microglia under our experimental conditions (Figure 1). Therefore, we used 50 μM taxifolin for subsequent experiments.

### 3.2. Effects of Taxifolin on the Intracellular ROS Levels Induced in an HG Environment

**Figure 2:**
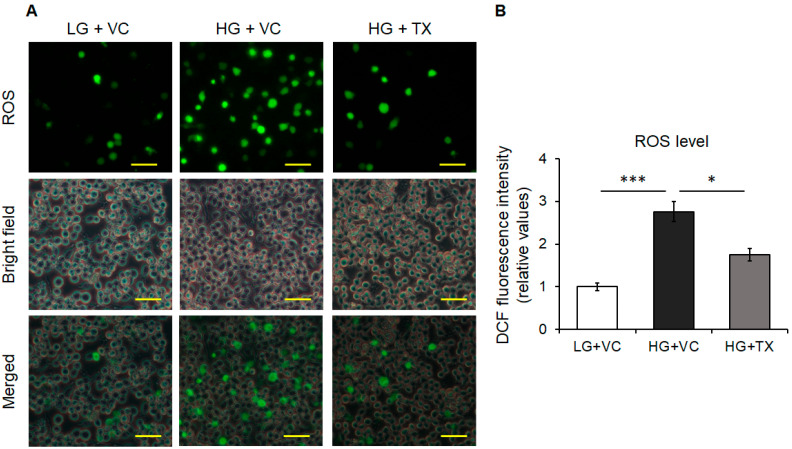
Effects of taxifolin (TX) on the intracellular reactive oxygen species (ROS) levels in BV-2 microglia. Cells were pretreated with 50 μM TX or the vehicle control (VC) in a low-glucose (LG) environment for 24 h, followed by stimulation with 75 mM high glucose (HG) or the VC for 24 h in the presence of TX or the VC. The intracellular ROS levels were examined using the DCFH-DA assay. Representative images (**A**) were obtained using a fluorescence microscope (upper panels, ROS detected using the fluorescence channel; middle panels, cell outlines obtained by the bright field channel; lower panels, merged images) (scale bars, 50 μm), and the ROS levels (**B**) were quantified upon measuring the fluorescence intensities. Data are expressed as the mean ± SEM (*n* = 3; three independent experiments were performed). * *p* < 0.05; *** *p* < 0.001.

Since an HG environment reportedly elevates the intracellular ROS levels [17,18,19,39], we examined the effects of taxifolin under this cytotoxic process. Our immunofluorescence analyses revealed that HG treatment significantly increased the intracellular ROS levels in BV-2 cells (Figure 2A,B), which is consistent with previous reports [17,18,19,39]. We observed that the ROS levels were significantly decreased via taxifolin treatment (Figure 2A,B). These results suggested that the antioxidant and radical scavenging activities of taxifolin inhibited the elevation of HG-induced intracellular ROSs in these cells.

### 3.3. Effects of Taxifolin on the Inflammatory Responses of BV-2 Microglia Triggered by HG Treatment

**Figure 3:**
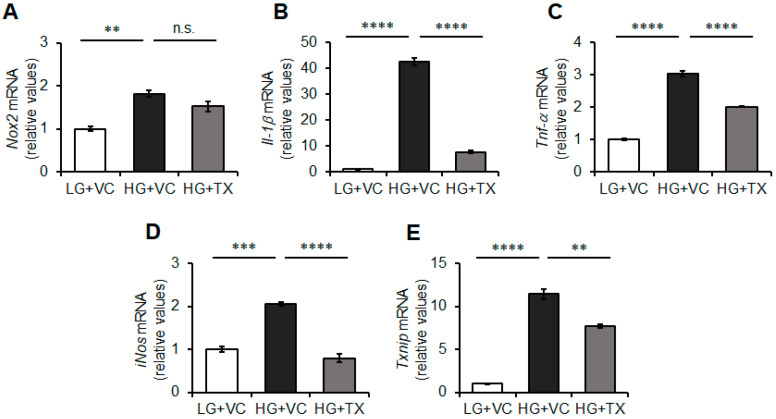

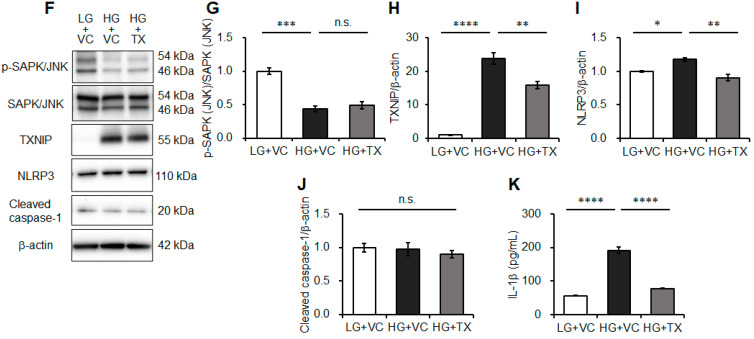
Effects of taxifolin (TX) on the high-glucose (HG)-induced inflammatory responses of BV-2 microglia. Cells were preincubated for 24 h with 50 μM TX or the vehicle control (VC) in a low-glucose (LG) environment, followed by treatment for 24 h with 75 mM HG or the VC in the presence of TX or the VC. The mRNA levels of NADPH oxidase (Nox) 2 (**A**), interleukin (IL)-1β (**B**), tumor necrosis factor-alpha (TNF-α) (**C**), inducible nitric oxide synthase (iNOS) (**D**), and thioredoxin-interacting protein (TXNIP) (**E**) were examined via quantitative RT-PCR and normalized to that of 18S. The quantity of proteins of interest was determined via Western blot (**F**) and densitometry (**G**–**J**). Fold changes are displayed relative to the VC (1.0) (**A**–**E**, **G**–**J**). IL-1β level (**K**) in the culture supernatant was measured using ELISA. Data are expressed as the mean ± SEM (*n* = 3; three independent experiments were performed). * *p* < 0.05; ** *p* < 0.01; *** *p* < 0.001; **** *p* < 0.0001; n.s., not significant.

HG-induced ROSs are reportedly implicated in triggering inflammatory responses in microglial cells [17]. Since taxifolin significantly reduced the intracellular ROS levels (Figure 2A,B), we subsequently investigated whether taxifolin was involved in attenuating the inflammatory mediators that were stimulated in an HG environment as well as the expression levels of ROS-generating enzymes Nox1, Nox2, and Nox4 [33]. Although Nox1 and Nox4 mRNAs were not detected under our experimental conditions, our results demonstrated that the gene expression levels of Nox2 were significantly elevated in HG-treated BV-2 microglia, whereas taxifolin did not significantly affect those of Nox2 (Figure 3A). Moreover, HG treatment upregulated the gene expression levels of the proinflammatory cytokines IL-1β and TNF-α, and taxifolin significantly reduced the expression levels of these genes in BV-2 microglia (Figure 3B,C). The gene expression levels of the inflammation-related factor iNOS [40] were also significantly decreased via taxifolin treatment (Figure 3D). Moreover, the HG environment elevated the expression of TXNIP, which was significantly suppressed by taxifolin treatment (Figure 3E). Regarding redox-sensitive SAPK/JNK MAPK [41], HG treatment for 24 h reduced the activation, and taxifolin did not exhibit significant effects (Figure 3F,G), thereby suggesting that this MAPK would respond to the early increase in oxidative stress [19]. Conversely, taxifolin significantly reduced the protein levels of TXNIP, NLRP3, and IL-1β in HG-treated BV-2 cells, whereas those of cleaved caspase-1 were not significantly affected by the HG environment or taxifolin treatment (Figure 3F,H–K). These results suggested that taxifolin exerts inhibitory effects on the HG-induced activation of BV-2 microglia by suppressing the TXNIP–NLRP3 axis through the antioxidant and radical scavenging activities in these cells.

## 4. Discussion

The present study provides the first evidence of the novel suppressive effects of taxifolin on microglial activation in an HG environment. Taxifolin exhibited anti-inflammatory effects on HG-stimulated microglia by suppressing the TXNIP–NLRP3 axis. Furthermore, the underlying mechanism of these effects has been implicated in the reduction in HG-induced ROS production. These findings contribute to the development of novel strategies for preventing neuroinflammation in diabetes.

Diabetes deleteriously affects microglial function and results in cognitive impairment in both mouse models [11,42,43] and patients with T2DM [12,13,14]. Moreover, an HG environment elevated the intracellular ROS levels and triggered inflammatory responses in the microglia, which were implicated in several pathways including NF-κB signaling [17,18], p38 and SAPK/JNK MAPK pathways [19], and NLRP3 inflammasome activation [20]. Since the HG environment significantly elevated the gene expression levels of Nox2 in this study, HG treatment would increase both cytosolic ROS production [44] and dysfunctional mitochondria-derived ROS levels [22,45] in the microglia. We further demonstrated that the HG environment increased the levels of TXNIP, an upstream positive regulator of NLRP3 inflammasome activation, in the microglia. Moreover, taxifolin inhibited the elevation of HG-induced TXNIP, which could subsequently reduce the NLRP3 levels, thereby suppressing IL-1β production. TXNIP expression is reportedly increased by the HG-induced elevation of ROS levels in the renal [22] and retinal cells [23]. Since taxifolin reduced the intracellular ROS levels in HG-treated microglia without a significant reduction in the gene expression levels of Nox2 in this study, the antioxidant and radical scavenging activities of taxifolin could reduce ROS levels and attenuate the activation of the TXNIP–NLRP3 axis, resulting in the suppression of IL-1β production. Conversely, the HG environment and taxifolin did not significantly affect the activation levels of caspase-1 in this study. Thus, these results suggest that the non-canonical TXNIP–NLRP3 axis is implicated in IL-1β production and that taxifolin could suppress the pathways in HG-stimulated microglia. In this respect, comparative studies on taxifolin and the other antioxidants as well as in silico studies to identify novel targets of taxifolin could help reveal the mechanisms of action specific to taxifolin. Since taxifolin has pleiotropic pharmacological actions, such as amyloid-β-biding properties [26,27], the unidentified bioactivities other than antioxidant and radical scavenging activities may also contribute to its suppressive effects on microglial activation. Although future studies are warranted to elucidate the mechanistic details, our findings demonstrated novel beneficial effects of taxifolin in suppressing neuroinflammation under diabetic conditions.

The pathological significance of ROS in HG-induced cytotoxicity has been addressed in detail in studies on keratinocytes. Elevated ROS levels in an HG environment cause mitochondrial dysfunction and mediate stress signaling cascades, which subsequently induce keratinocyte apoptosis [46]. Notably, exposure to HG results in a decrease in the intracellular antioxidant factors, such as superoxide dismutase, thereby resulting in the progression of oxidative stress [46]. Thus, maintaining antioxidant capacity is warranted to prevent the vicious cycle of HG-augmented ROS production, reductions in antioxidant factors, and the exacerbation of ROS-related cytotoxicity; the present study demonstrated that taxifolin suppresses inflammatory responses by reducing the intracellular ROS levels in HG-stimulated microglia. Moreover, we recently demonstrated the beneficial effects of orally administered taxifolin on cognitive preservation in patients with mild cognitive impairment or mild dementia [31]. Therefore, the findings of this study highlight that taxifolin can be implemented in clinical practice to prevent and improve ROS-related pathologies in various diseases as well as neuroinflammation in diabetic conditions, thereby further emphasizing the significance of future clinical trials.

In conclusion, this study demonstrated the novel functional significance of taxifolin in suppressing microglial inflammatory responses in an HG environment. The antioxidant and radical scavenging activities of taxifolin reduced HG-induced ROS production, which further suppressed the TXNIP–NLRP3 axis, thereby inhibiting IL-1β production. Since taxifolin also has beneficial effects on glucose metabolism under diabetic conditions [30], it can indirectly suppress cognitive impairment by improving metabolism; therefore, the indirect effects and direct impact of taxifolin on the microglia can synergistically prevent and/or improve diabetes-related cognitive impairment. Future preclinical and clinical studies to address the in vivo effects of taxifolin on neuroinflammation in diabetic conditions would be helpful in developing novel strategies to prevent diabetes-related cognitive impairment.

## 5. Conclusions

We demonstrated novel suppressive effects of taxifolin on neuroinflammation under diabetic conditions; taxifolin reduced the intracellular ROS levels and attenuated the activation of the TXNIP–NLRP3 axis, thereby suppressing IL-1β production in HG-treated microglia. These findings could contribute to the development of innovative strategies for preventing and treating diabetes-related cognitive impairment.

## Data Availability

The data presented in this study are available from the corresponding authors upon reasonable request.
